# Combination chemoradiotherapy with temozolomide, vincristine, and interferon-β might improve outcomes regardless of O6-methyl-guanine-DNA-methyltransferase (*MGMT*) promoter methylation status in newly glioblastoma

**DOI:** 10.1186/s12885-021-08592-z

**Published:** 2021-07-28

**Authors:** Kenichiro Asano, Toshio Fumoto, Masashi Matsuzaka, Seiko Hasegawa, Naoya Suzuki, Kenichi Akasaka, Kosuke Katayama, Akihisa Kamataki, Akira Kurose, Hiroki Ohkuma

**Affiliations:** 1 Department of Neurosurgery, Hirosaki University Graduate School of Medicine, 5 Zaifu-cho, Hirosaki, Aomori 036-8562 Japan; 2 Clinical Research Support Center, Hirosaki University Hospital, 53 Hon-cho, Hirosaki, Aomori 036-8563 Japan; 3 Department of Medical Informatics, Hirosaki University Hospital, 53 Hon-cho, Hirosaki, Aomori 036-8563 Japan; 4 Department of Neurosurgery, Kuroishi General Hospital, 1-70 Kitami-cho, Kuroishi, Aomori 036-0541 Japan; 5 Department of Neurosurgery, Towada City Hospital, 8-14 Nishi-Jyuniban-cho, Towada, Aomori 034-0093 Japan; 6 Department of Anatomic Pathology, Hirosaki University Graduate School of Medicine, 53 Honcho, Hirosaki, Aomori 036-8563 Japan

**Keywords:** Newly glioblastoma, Combination therapy, Temozolomide, Interferon-β, *MGMT*

## Abstract

**Background:**

This investigator-initiated, open-label, single-arm, single-institute study was conducted to investigate the effectiveness of induction combination chemoradiotherapy and long-term maintenance therapy with temozolomide (TMZ) plus interferon (IFN)-β for glioblastoma.

**Methods:**

The initial induction combination chemoradiotherapy comprised radiotherapy plus TMZ plus vincristine plus IFN-β. Maintenance chemotherapy comprised monthly TMZ, continued for 24–50 cycles, plus weekly IFN-β continued for as long as possible. The primary endpoint was 2-year overall survival (2y-OS). The study protocol was to be considered valid if the expected 2y-OS was over 38% and the lower limit of the 95% confidence interval (CI) was no less than 31.7% compared with historical controls, using Kaplan-Meier methods. Secondary endpoints were median progression-free survival (mPFS), median OS (mOS), 5-year OS rate (5y-OS), and mPFS and mOS classified according to *MGMT* promoter methylation status.

**Results:**

Forty-seven patients were analyzed. The 2y-OS was 40.7% (95%CI, 27.5–55.4%). The mPFS and mOS were 11.0 months and 18.0 months, respectively, and 5y-OS was 20.3% (95%CI, 10.9–34.6%). The mPFS in groups with and without *MGMT* promoter methylation in the tumor was 10.0 months and 11.0 months (*p* = 0.59), respectively, and mOS was 24.0 months and 18.0 months (*p* = 0.88), respectively. The frequency of grade 3/4 neutropenia was 19.1%.

**Conclusions:**

The 2y-OS with induction multidrug combination chemoradiotherapy and long-term maintenance therapy comprising TMZ plus IFN-β tended to exceed that of historical controls, but the lower limit of the 95%CI was below 31.7%. Although the number of cases was small, this protocol may rule out *MGMT* promoter methylation status as a prognostic factor.

**Trial registration:**

University Hospital Medical Information Network (number UMIN000040599).

## Background

Glioblastoma (GBM) remains an incurable disease. The reported median overall survival (mOS) and 2-year OS rate (2y-OS) in patients receiving treatment according to the Stupp protocol (temozolomide [TMZ] plus radiotherapy [RT]), which is regarded as the international standard of care, were 14.6 months and 26.5%, respectively [1]. That protocol represented a great paradigm shift in chemotherapy as compared to the treatment with alkylating agents used prior to the introduction of TMZ. The introduction of TMZ has significantly improved treatment outcomes. However, follow-up continuing beyond 2 years has shown that the 5-year OS rate (5y-OS) in patients receiving RT plus TMZ was only 9.8%, not significantly different from results obtained under previous conventional regimens [2]. Also, according to a systematic review by Tykocki et al. [3], the 10-year OS rate is a dismal 0.71%. Complete cure of GBM thus still seems to be a far-fetched goal. In addition, the incidence of GBM has not decreased. According to the Brain Tumor Registry of Japan (2005–2008) [4], about 1000 patients are newly diagnosed with GBM in Japan each year. Improving treatment outcomes for GBM is thus an urgent issue worldwide.

The primary treatment strategy for patients with newly diagnosed GBM is resection; that is, removal of as much of the tumor as possible [5, 6], followed by combined RT plus TMZ therapy. Alternatively, the latest treatment method can be used: a combination of the Novo Tumor Treating Fields system plus TMZ maintenance treatment [7]. However, tumor O6-methyl-guanine-DNA-methyltransferase (*MGMT)* promoter methylation status is considered a prognostic factor, and an obstacle that cannot be overcome by the initial induction therapy and 6 subsequent cycles (C) of maintenance treatment with TMZ [8, 9]. *MGMT* promoter methylation status has also been considered a prognostic factor for GBM in recent meta-analyses [10, 11].

PCV multidrug combination therapy with procarbazine, Lomustine (CCNU) and vincristine was widely used for the treatment of high-grade glioma (HGG) in Western countries [12]. Nimustine (ACNU) was developed in Japan [13], and has been used in this country as a community standard therapy in synchronous chemotherapy (RT + ACNU+VCR) [14] and IAR therapy (RT+ ACNU+IFB-β) [15, 16] for HGG.

Treatment with VCR has been reported to induce a high accumulation of tumor cells during the highly radiosensitive G2-M phase of the cell cycle. In addition, a flow cytometric study of the growth pattern revealed that about 10% of cells in the mitotic phase were in the G2-M phase, suggesting the promise of combination therapy with VCR [17]. Interferon (IFN)-β is classified as a type I IFN and was discovered as a cytokine with antiviral activity. IFN-β has since been shown to exert various biological activities, such as immunostimulatory activity, angiogenesis-inhibitory activity, antiproliferative activity, and anti-tumor activity mediated by induction of apoptosis [18]. IFN-β was found to be useful in the treatment of not only high-grade glioma (HGG) [15, 16] but also low-grade glioma, with minor adverse drug reactions and a high response rate [19]. Clinical trials have also been conducted overseas for recurrent HGG, showing progression-free survival (PFS) of 23 weeks and a 23% partial response rate [20].

Utilizing these advantages, Aoki et al. [21] conducted a phase II study of combined chemoradiotherapy; that is, RT with ACNU + carboplatin + VCR + IFN-β, in patients with newly diagnosed GBM, with the expectation of additive and synergistic effects of the drugs. They reported a PFS of 10 months, and an OS of 16 months. We also investigated the clinical usefulness of RT administered with the combination regimen of ACNU + VCR (unpublished data). Meanwhile, Stupp et al. reported the results of treatment with TMZ for newly diagnosed cases of GBM [1]. The study reported by Stupp et al. [1] was definitely an epoch-making event, demonstrating significant prolongation of OS by a single agent in patients with newly diagnosed GBM. However, the results were still far from satisfactory. By that time, basic experiments had revealed TP53-mediated inactivation of *MGMT* by IFN-β [22]. We therefore developed a new toxic multidrug combination by replacing the conventionally used ACNU with TMZ and adding IFN-β, resulting in TMZ + VCR + IFN-β.

We have therefore devised a new treatment strategy involving the use of a multidrug combination, including IFN-β in the initial induction therapy administered with RT, followed by maintenance therapy with TMZ and IFN-β administered for as long as possible, aimed at depleting tumor MGMT. This post-hoc analysis investigated the efficacy and safety of our proposed treatment, which we expected to yield better treatment outcomes than the conventional regimen.

## Methods

### Patients

Inclusion criteria for this study were as follows: 1) pathologically confirmed newly diagnosed GBM (including giant cell glioblastoma or gliosarcoma) as defined by the World Health Organization classification of tumors 2007 (IARC 4th edition); 2) age, 16–80 years; 3) tumor located without involvement of the optic nerve, hypothalamus, or ventricles (containing most of the tumor mass, but not involving the brainstem), and without cerebrospinal fluid dissemination at initial diagnosis; 4) no history of malignant tumors, and no previous or current history of chemotherapy; 5) no previous history of RT; 6) no multiple primary cancers; 7) Eastern Cooperative Oncology Group performance scale (ECOG-PS), 0–2, or 3 in the presence of neurological symptoms; 8) adequate organ functions; 9) no serious infectious diseases; 10) patient suitable to receive treatment within 28 days of undergoing surgery; 11) regardless of the extent of surgical resection (biopsy is also acceptable); and 12) provision of informed consent for participation in the study by the patient or their legal representative.

### Treatments

The initial induction therapy was RT and concomitant chemotherapy with TMZ + VCR + IFN-β. RT (at 2.0 Gy/fr/day, 30 fr) and TMZ (75 mg/m^2^/day) were started simultaneously on Day 1. Oral TMZ was administered daily before breakfast and continued for 42 days. Up to 45 days of TMZ treatment was accepted, allowing for holidays and rest days for maintenance of the RT equipment. On days 2 and 3, vincristine (VCR) was administered by intravenous injection at 0.6 mg/m^2^. On day 5, a thrice-weekly regimen of IFN-β was started; the drug was administered by intravenous drip infusion at 3 MU/body over 1 h. After completion of the initial induction therapy, study patients were followed-up for 28 days until the start of maintenance therapy. RT comprised fractionated focal irradiation administered at a fractional dose of 2 Gy given once a day, 5 days a week (Monday through Friday) for a period of 6 weeks, to a total dose of 60 Gy. The radiation treatment plan was the same as applied in the Stupp protocol and JCOG0911 [1, 23].

Maintenance therapy was started with oral TMZ (150 mg/m^2^/day, for 5 days) administered before breakfast after day 28 when the patient met all blood test criteria. The permissible treatment interruption period for TMZ was set at 23 days. Dose-increases of TMZ after the second cycle from 150 to 200 mg/m^2^/day (for 5 days) were allowed in patients who met the blood test criteria, but no further dose increase was allowed from the third cycle onward. If the next cycle could not be started within 63 days from the start of the previous cycle, or if G4 neutropenia or G3/4 thrombopenia was present in the previous cycle, TMZ was dosed down (from 200 to 150 mg/m^2^/day, 5 days; 150 to 100 mg/m^2^/day, 5 days). If it was longer than that, it was canceled. TMZ treatment was continued for at least 24 cycles. Even after that, whether the patient was willing to continue treatment was assessed at 30 and 40 cycles, and continuation of TMZ treatment was allowed up to 50 cycles. IFN-β was administered at 3 MU/body, once weekly. Dose adjustment of IFN-β within 1–3 MU/ body was allowed, taking into account body weight and myelosuppression. IFN-β treatment was continued for at least 2 years, and for as long as possible thereafter. Treatment after recurrence was not specified in the protocol, and patients were given the liberty to choose any course of treatment.

### Study design

This study was an investigator-initiated, open-label, single-arm, single-institute trial. A threshold 2y-OS of 33% was set for this study, based on the study results reported by Stupp et al. [1], other study results [14, 15, 21], and our previous study (unpublished data) in which the 2y-OS for RT + ACNU+VCR was 33%. We used IFN-β as add-on therapy in our proposed treatment, as the so-called toxic new regimen. Also, taking into account patient burden and medical expenses, the survival rate in patients receiving the combined chemoradiotherapy (RT with TMZ + VCR+ IFN-β) should exceed that in historical controls by ≥5%. Therefore, for the 2y-OS threshold set at 33% for this therapy, the expected 2y-OS was set at 38% using the Kaplan-Meier method, with a registration period covering 5 years and a follow-up of 5 years. The one-sided significance level of testing the results of the main analysis was set at 10%, because the population of our hospital is small (1.2 million), the disease is rare and shows poor prognosis, and little variation was seen in cases due to the performance of almost all interventions by a single surgeon. The number of eligible patients necessary to obtain a statistical power of 80% was calculated as minimum 44 according to the formula of Simon et al. [24]. With allowance for ineligible patients, the number of patients to be enrolled in this study was set at 50.

This study was conducted in compliance with the Declaration of Helsinki and guidelines on Good Clinical Practice, and was conducted with the approval of The Committee of Medical Ethics at the University Graduate School of Medicine, Hirosaki, Japan (approval no. 2007–142). After the confirmation of histopathological diagnosis, patients who provided informed consent were enrolled in the Neurosurgical Clinical Study Registry of Hirosaki University Hospital, and trial treatment was started within 28 days after surgery.

### Statistical analysis

The primary endpoint was the 2y-OS. Secondary endpoints were progression-free survival (PFS), 5y-OS, overall survival (OS), and PFS and OS classified according to tumor *MGMT* promoter methylation status, and the frequency/nature of adverse events. PFS was defined as the period from the date of surgery to the date of MRI confirmation of recurrence. OS was defined as the period from the date of surgery to the date of death or last date of confirmed survival. Adverse events were coded according to the Common Terminology Criteria for Adverse Events (CTCAE) version 3.0. *MGMT* promoter methylation status was determined by methylation-specific polymerase chain reaction (MS-PCR) using surgical specimens, as described later [8, 25]. The extent of surgical resection was determined from findings on MRI performed within 72 h after surgery. In addition, tumor immunostaining was performed after surgery to detect IDH1^R132H^ mutations (Anti-IDH1^R132H^ antibody [H09]; DIANOVA GmbH, Hamburg, Germany).

In the main analysis, the efficacy of our proposed treatment was determined based on an expected 2y-OS of this study being greater than that of historical controls. In other words, if the result from Kaplan-Meier analyses was greater than or equal to 38% of the expected 2y-OS (26.5%, as the 2y-OS of the Stupp trial [1]), and the lower limit of the 95% confidence interval (CI) of our study was no less than 31.7%, as the result from the study by Stupp et al. [1] (i.e., the 95%CI included the value of 31.7%), the method was to be considered effective. OS and PFS were examined using Kaplan-Meier methods. Determination of the OS and PFS according to *MGMT* promoter methylation status was performed by log-rank test. All statistical analyses were performed on a Mac OSX version 10.14.5 operating system, using JMP version 14 statistical software (SAS Institute, Cary, NC).

### MS-PCR for determining MGMT promoter methylation status

DNA extraction from formalin-fixed, paraffin-embedded tissue sections and subsequent bisulfite conversion were performed using the Methylamp Whole Cell Bisulfite Modification Kit, in accordance with the instructions from the manufacturer (EPIGENTEK, Farmingdale, NY). PCR was performed using ZymoTaq PreMix (ZYMO RESEARCH, Irvine, CA) and a C1000 Thermal Cycler (Bio-Rad, Hercules, CA). Amplified products were separated on 4% agarose gels, stained with ethidium bromide, and visualized using the Gel Doc EZ Imager (Bio-Rad). For each PCR reaction, a Human Methylated & Non-methylated DNA Set (ZYMO RESEARCH, Irvine, CA) was used as the control. These reaction conditions and the sequences of the PCR primers were as previously described in the literature [8, 25].

## Results

### Patients

**Fig. 1:**
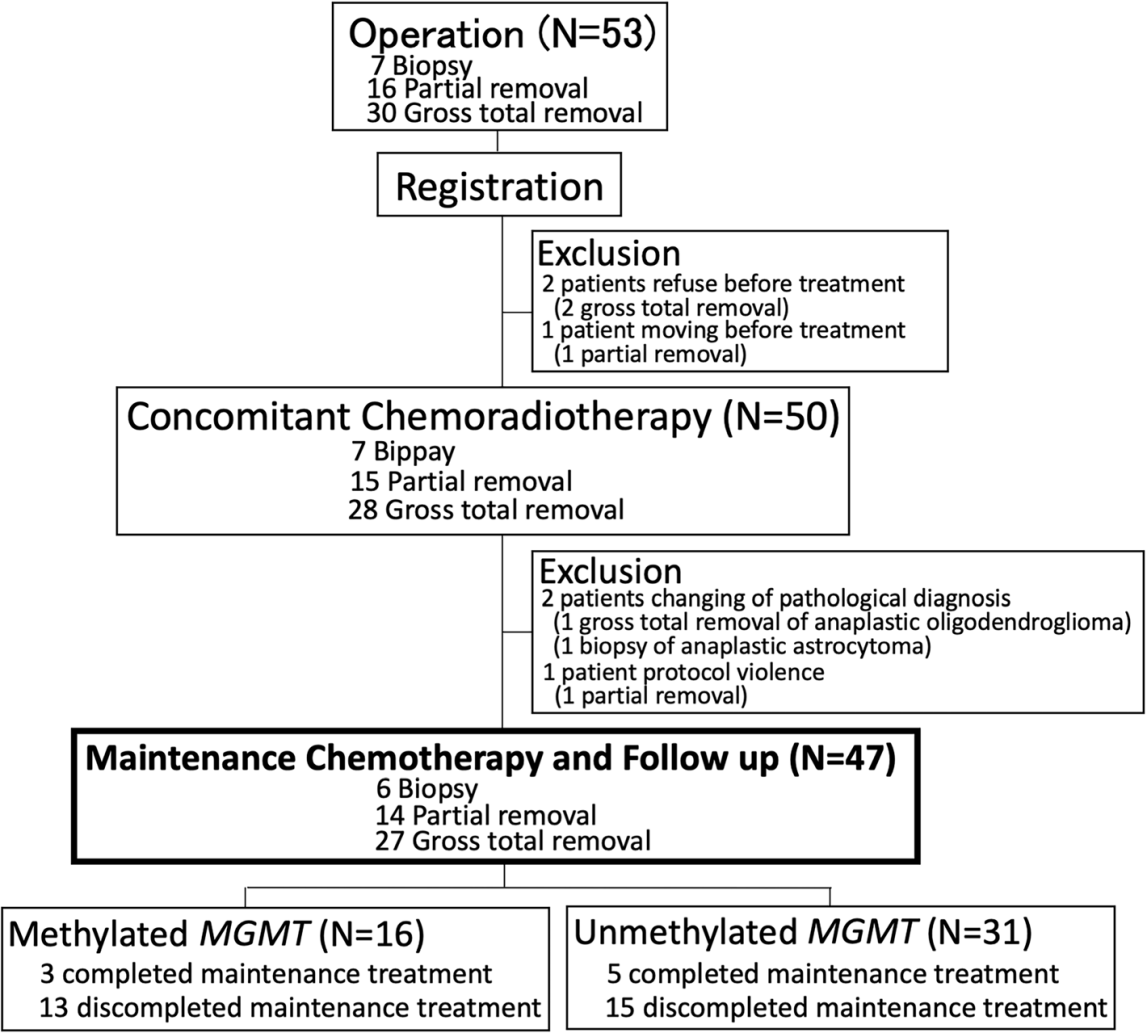
Recruitment and inclusion of patients in this study

Patients were enrolled in this study between April 2008 and March 2013. Originally, 53 patients were enrolled to the study and a final total of 47 patients thus received the protocol treatment (Fig. 1).

The median age of patients was 62 years (range, 16–80 years), and 10 patients (21.3%) were elderly (≥71 years old). Twenty patients (42.6%) had an ECOG-PS of 2, indicating that many patients were in a serious condition. Total tumor resection was achieved in 27 patients (57.4%). Immunostaining of the resected tumor revealed wild-type *IDH1*^R132H^ in 41 patients (87.2%) and a mutant gene in 6 patients (12.8%). *MGMT* promoter methylation status as determined by MS-PCR was rated as “methylated” in 16 patients (34.0%) and “unmethylated” in 31 patients (66.0%). No dropouts or treatment discontinuations due to emergence of adverse events to the initial induction therapy were encountered. The median number of TMZ maintenance therapy cycles administered was 13 (range, 3–50), and the median number of IFN-β maintenance therapy cycles administered was 51 (range, 10–328) (Table 1).

**Table 1:** Baseline characteristics and treatment details of patients

Characteristic	The number of patients (*N* = 47)
Age	–
Median (IQR), Range	62 (54, 69), 16–80
Age ≤ 70	37 (78.7%)
Age ≥ 71	10 (21.3%)
Sex	–
M: F	21:26
ECOG performance status	–
0	13 (27.7)
1	14 (29.8)
2	20 (42.6)
3	0 (0.0)
Operation removal rate	–
Biopsy	6 (12.8)
Partial resection	14 (29.8)
Gross total removal	27 (57.4)
Pathological diagnosis	–
Glioblastoma	43 (91.5)
Giant cell glioblastoma	3 (6.4)
Gliosarcoma	1 (2.1)
IDH status (IDH1^R132H^)	–
Wild	41 (87.2)
Mutant	6 (12.8)
MGMT status	–
Methylated	16 (34.0)
Unmethylated	31 (66.0)
Concomitant induction phase	–
Complete protocol	47 (100)
Incomplete protocol	0 (0.0)
Maintenance phase	–
Complete protocol of TMZ 24C + IFN-β 96C	10 (21.3)
Complete protocol of TMZ 50C + IFN-β 200C	5 (10.6)
Maintenance cycles of TMZ (Median (IQR) Range)	13 (8, 23), 3–50
Maintenance cycles of IFN-β (Median (IQR) Range)	51 (32, 86), 10–328

Data are number (%).

### Clinical course

Six of the 47 patients remained alive, 39 had died, and 2 were lost to follow-up. Of the 39 patients who died, 2 died of comorbid diseases, including myocardial infarction and acute aortic dissection, and the remaining 37 died of the tumor or tumor-related complications. Under this protocol, 10 patients (21.3%) continued TMZ maintenance therapy for 2 years without recurrence, and 5 patients (10.6%) continued TMZ maintenance therapy for 5 years without recurrence (Table 1). Six patients (12.8%) discontinued after 50 cycles of TMZ, including 5 patients who completed 5 years of relapse-free treatment and 1 patient who relapsed during treatment and continued to use TMZ.

**Fig. 2:**
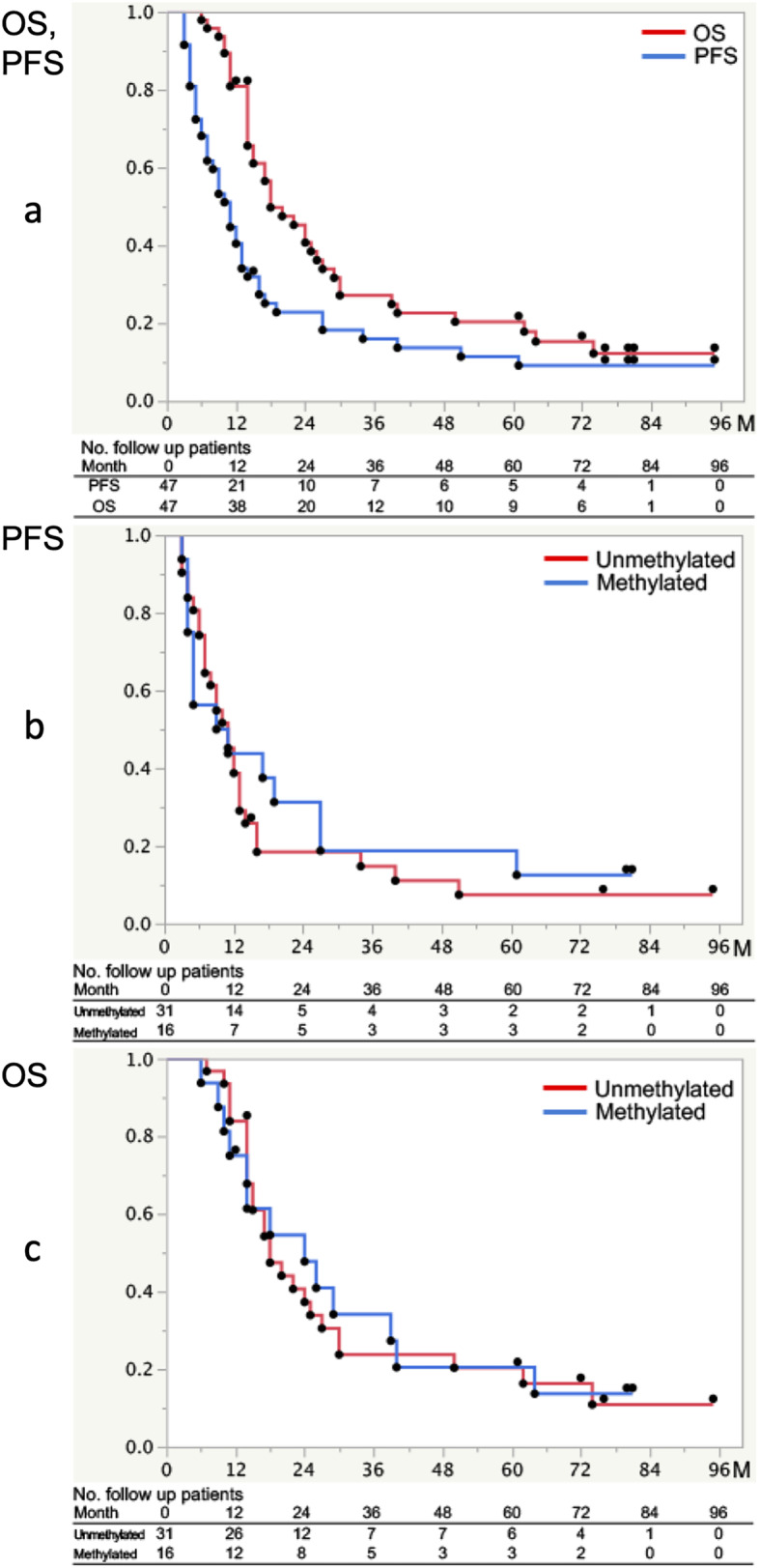
Kaplan-Meier curves for PFS and OS. **a** Kaplan-Meier estimates of progression-free survival (PFS) and overall survival (OS). PFS and OS were determined from date of registration until either tumor progression or last follow-up (censored patients), or until death or last follow-up (censored patients), respectively. **b** Kaplan-Meier estimates of PFS, according to *MGMT* promoter methylation status. PFS was 11.0 months (95%CI, 7.0–13.0 months) for the 31 patients of the unmethylated group and 10.0 months (95%CI, 4.0–27.0 months) for the 16 patients of the methylated group. Log-rank testing revealed no significant difference (*p* = 0.59). **c** Kaplan-Meier estimates of OS, according to *MGMT* promoter methylation status. OS was 18.0 months (95%CI, 14.0–27.0 months) in the unmethylated group and 24.0 months (95%CI, 11.0–40.0 months) in the methylated group. Log-rank testing showed no significant difference (*p* = 0.88)

The 2-year PFS rate (2y-PFS) was 22.8% (95%CI, 12.9–37.1%). The primary endpoint, 2y-OS, was 40.7% (95%CI, 27.5–55.4%). The 2y-OS was 7.7% higher than that recorded for the in-house treatment (unpublished data). In addition, 2y-OS in this study exceeded the expected 2y-OS of 38%, but the lower limit of the 95%CI was below 31.7%. The 5-year PFS rate (5y-PFS) was 11.4% (95%CI, 4.9–24.5%), and the 5y-OS was 20.3% (95%CI, 10.9–34.6%). Median PFS (mPFS) was 11.0 months, and mOS was 18.0 months (Fig. 2a, Table 2).

**Table 2:** Progression-free survival (PFS) and overall survival (OS)

Variable	PFS/OS (M / %)	95%CI
Median PFS (M)	11.0	7.0–13.0
At 1 year (%)	40.4	27.5–54.9
At 2 year (%)	22.8	12.9–37.1
At 3 year (%)	15.9	7.9–29.7
At 4 year (%)	13.6	6.4–27.1
At 5 year (%)	11.4	4.9–24.5
Median OS (M)	18.0	15.0–26.0
At 1 year (%)	80.8	67.1–89.7
At 2 year (%)	40.7	27.5–55.4
At 3 year (%)	27.1	16.1–41.9
At 4 year (%)	22.6	12.6–37.1
At 5 year (%)	20.3	10.9–34.6

*CI* Confidence interval

### PFS and OS according to *MGMT* promoter methylation status

Examined by *MGMT* promoter methylation status as determined by MS-PCR, mPFS was 11.0 months (95%CI, 7.0–13.0 months) in the 31 patients of the unmethylated group and 10.0 months (95%CI, 4.0–27.0 months) in the 16 patients of the methylated group. Log-rank test revealed no significant difference (*p* = 0.59) in PFS between groups. The mOS was 18.0 months (95%CI, 14.0–27.0 months) in the unmethylated group and 24.0 months (95%CI, 11.0–40.0 months) in the methylated group. Log-rank test showed no significant difference (*p* = 0.88) in OS between groups (Fig. 2b, c).

### Post-protocol treatments

With regard to treatment course after recurrence, which the patients were given the liberty to choose, the majority (26 patients; 61.9%) opted to continue maintenance therapy with TMZ and IFN-β. Five patients underwent additional surgery, 12 underwent additional stereotactic radiosurgery, and 2 received bevacizumab (BEV). Most patients (40 patients; 95.2%) continued combination therapy with TMZ and IFN-β (Table 3).

**Table 3:** Second-line treatments after first recurrence

Treatment Regimen	The Number of Patients *N* = 42 (%)
Same as protocol of TMZ + IFN-β	26 (61.9)
TMZ + IFN-β + SRS	8 (19.0)
TMZ + IFN-β + SRS + BEV	1 (2.4)
TMZ + IFN-β + SRS + Surgery	3 (7.1)
TMZ + IFN-β + Surgery+BEV	1 (2.4)
TMZ + INF-β + Surgery	1 (2.4)
Subtotal (TMZ + IFN-β)	40 (95.2)
Surgery alone	1 (2.4)
Best supportive care	1 (2.4)

*SRS* Stereotactic radiosurgery, *BEV* Bevacizumab

### Adverse events

Table 4 shows adverse events observed throughout the initial induction therapy and maintenance therapy periods. One patient developed IFN-associated retinopathy (CTCAE G3) in the maintenance phase. Although this patient, who had underlying hypertension and diabetes mellitus, was a high-risk patient, the causal relationship between the event and IFN-β was assessed as “probable,” and the patient therefore stopped IFN-β. Three patients developed CTCAE grade 1 peripheral sensory neuropathy, which was considered to have been caused by VCR, and the causal relationship with the drug was assessed as “probable.” In addition, 2 patients developed CTCAE grade 3 hydrocephalus, with the causal relationship to the treatment assessed as “unlikely”. As common toxicities, CTCAE grade 1–4 lymphopenia occurred in all patients, and grade 3/4 lymphopenia was noted in 87.3% of patients. Grade 3/4 neutropenia occurred in 19.1% of patients, while no patients developed febrile neutropenia. The incidence of thrombocytopenia was also high, at 72.5% overall, with 8.6% developing grade 3/4 thrombocytopenia. Anorexia also occurred at a relatively high incidence of 57.4% overall, with 8.5% developing grade 3/4 anorexia. Incidence of constipation was high, at 80.8%, but was controllable with medications in most cases.

**Table 4:** Adverse events

	Grade 1 (%)	Grade 2 (%)	Grade 3 (%)	Grade 4 (%)	Grade 3 + 4%
Hematological					
Anemia	14 (29.8)	19 (40.4)	0	0	0.0
Neutropenia	6 (12.8)	18 (38.3)	8 (17.0)	1 (2.1)	19.1
Lymphopenia	3 (6.4)	3 (6.4)	28 (59.6)	13 (27.7)	87.3
Thrombopenia	21 (44.7)	9 (19.2)	2 (4.3)	2 (4.3)	8.6
Non-hematological					
Nausea	7 (14.9)	5 (10.6)	2 (4.3)	0	4.3
Vomiting	4 (8.5)	4 (8.5)	1 (2.1)	0	2.1
Anorexia	16 (34.0)	7 (14.9)	4 (8.5)	0	8.5
Constipation	12 (25.5)	26 (55.3)	0	0	0.0
ALT elevation	16 (34.0)	10 (21.3)	2 (4.3)	0	4.3
Hyponatremia	26 (55.3)	3 (6.4)	2 (4.3)	0	4.3
Hyperpotassemia	11 (23.4)	1 (2.1)	1 (2.1)	0	2.1
Skin rush	3 (6.4)	4 (8.5)	0	0	0.0
Fever	15 (31.9)	0	0	0	0.0
Febrile neutropenia	–	–	0	0	0.0
Others	3 (6.4)	0	3 (6.4)	0	6.4

Others: Interferon-associated retinopathy (G3) 1 case, hydrocephalus(G3) 2 cases, and Peripheral sensory neuropathy(G1) 3 cases

## Discussions

The primary endpoint of 2y-OS in the present trial of a new treatment regimen we developed was 40.7% (95%CI 27.5–55.4%). This was higher than the 33% from our unpublished in-house data, the expected 2y-OS of 38%, and the 2y-OS of 26.5% reported by Stupp et al. [1], but the lower limit of the 95%CI for this study was below 31.7%, so the result was not valid. In addition, mPFS and mOS, set as the secondary endpoints, were 11.0 and 18.0 months, respectively, both of which were superior to the results reported [1]. The 5y-OS was also higher (20.3%; 95%CI 10.9–34.6%) than the reported result of the follow-up study [2], but the lower limit of the 95%CI for this study was again insufficient.

Nevertheless, the mPFS and mOS of 11.0 and 18.0 months, respectively, which were longer than those reported previously (6.9 months and 14.6 months, respectively) [1], did not represent significant improvements. This could be because the entry criteria for our study population were less stringent, to better represent actual clinical settings. For example, the study population included elderly people up to 80 years old, and no limit was placed on tumor size, so inclusion of even very large tumors with volume ≥ 100 mL was permitted. The study population thus included patients with poor preoperative PS and patient ineligible for total resection (gross total removal rate, 57.4%). In other words, the study population included patients more likely to die earlier. However, although not significant, our results for survival and duration (2y-OS-5y-OS, PFS, OS, etc.) tended to be higher than those reported by Stupp et al. [1, 2]. Another feature of this study was that the survival rate after more than 2 years remained relatively high. In addition, a number of studies in addition to the present investigation have concluded that prognosis is unaffected by the presence or absence of MGMT methylation status, which has often been considered a prognostic factor [26–30], but this study is one of them. We discuss the potential reasons below.

First, one reason could be our continuation of maintenance therapy with TMZ for as long as possible. Our cohort included 6 responders (12.8%), with the inclusion of 1 case with recurrence, and treatment in these cases was discontinued after 50 cycles of TMZ. In addition, 40 patients (95.2%) received TMZ plus IFN-β therapy in combination with BEV, surgery or stereotactic radiosurgery, etc., even after the development of recurrence. In actual clinical practice in Japan, 6 treatment cycles of TMZ as specified in the protocol reported by Stupp et al. [1] is not sufficient, and TMZ treatment is actually continued for longer in many cases. In the literature, one report has described administration of TMZ for 101 cycles [31], and another indicated that long-term treatment with TMZ until progression is more cost-effective than a treatment protocol that recommends completion of TMZ treatment after 6 cycles [32]. Many publications have discussed the efficacy of long-term treatment [31, 33–40]. A meta-analysis has indicated that long-term treatment is more beneficial in terms of both OS and PFS [41]. In addition, according to one study, MGMT-mediated TMZ resistance may be attenuated by continuous therapy [42]. Certainly, long-term administration of TMZ would enhance the accumulation of TMZ in the body, and may have the same significance as the use of dose-dense TMZ, which depletes MGMT. On the other hand, some studies reporting on the benefits of long-term treatment with TMZ have also indicated that prognosis varies depending on tumor *MGMT* promoter methylation status [31, 33–35]. We cannot definitively conclude that long-term treatment with TMZ in our study resulted in good prognosis regardless of tumor *MGMT* promoter methylation status. Of course, TMZ could be considered ineffective for patients with unmethylated MGMT. Hegi et al. also reported that combined TMZ plus RT in the unmethylated group yielded a significantly prolonged PFS as compared to RT alone, and in the unmethylated group, combined TMZ plus RT as compared to RT alone tended to prolong OS, although those differences were not significant [8]. Furthermore, MGMT expression is heterogeneous and varies depending on the assay, and the site and timing of the assay [43, 44]. Suggesting that long-term treatment with TMZ contributes to improvement of PFS and OS may thus be acceptable.

No data have since demonstrating the efficacy of VCR alone in patients with brain tumors [45]. However, VCR is always used together with other antitumor agents, to deliver synchronous chemotherapy [14] or to obtain additive or synergistic effects [21, 46–48]. A meta-analysis revealed that VCR can become an antagonist for ACNU, BCNU, and cytosine arabinoside (Ara-C), and can synergize with CCNU, procarbazine, and cyclophosphamide. Only limited reports have described the use of VCR in combination with TMZ, but it is not an antagonist [45]. It remains necessary to accumulate more cases in the future, but at present, it appears that combined therapy with TMZ may contribute to the prolongation of PFS and OS.

Why then did the results of this study suggest that OS was unrelated to *MGMT* promoter methylation status? As mentioned above, continued treatment with IFN-β could be one reason. The INTEGRA study [49] was conducted using combined TMZ plus IFN-β therapy, based on the results of basic experiments reported by Natsume et al. [22], aimed at depleting MGMT, followed by the phase-II JOCG0911 study [23]. The add-on efficacy of IFN-β was denied in this JCOG0911 study, because no superiority of TMZ plus IFN-β could be established. However, a definitive difference from our study was seen in the dose of IFN-β. While the dose used for induction therapy was the same, that used for maintenance therapy was 4-fold higher in our study, leading to differences in continuing treatment. This may be one reason for the superior results in our study. Since the report from Hegi et al. [8], *MGMT* promoter methylation status has generally been viewed a prognostic marker, and meta-analyses have supported this stance [10, 11]. In recent years, however, *MGMT* promoter methylation status has been suggested to not be the only prognostic marker. Sex differences [28] and Asian and Serb ethnicity are not associated with *MGMT* promoter methylation status and prognosis [26, 27, 29]. A retrospective study from a large single institute also found no relationship with *MGMT* promoter methylation status [30]. GBM therefore shows complex biological diversity, and some diversity may not be explained only by the addition of extended maintenance TMZ, VCR and IFN-β. Of course, the absolute number of patients in this study was low and may have resulted in insufficient statistical power.

In addition, continued treatment with TMZ causes lymphopenia in most cases. Likewise in this study, lymphopenia was observed in all patients, reaching grade 3/4 in 87.3%. Lymphopenia caused by TMZ is said to be characterized by depletion of a particularly high proportion of CD4-positive helper T cells [50]. If helper T cells disappear, the response of CD8-positive killer T cells also disappears, probably resulting in decreased antitumor effects. However, IFN-β can compensate for this, exerting cytocidal activity and providing immunotherapeutic advantages [18]. In other words, during the first 2 years or so of treatment, the main effect is produced by anticancer agents, such as TMZ, which has cytocidal effects. Thereafter, IFN-β may exert immune antitumor actions. However, this also remains speculative.

Verification for the above findings of dosage and immunology requires a well-designed prospective study based on patient conditions under certain statuses in collaborative work with other institutions, and we have to identify those populations in which IFN-β is most effective by performing sub-analyses for each *MGMT* methylation status and other biomodulators.

With regard to adverse drug reactions, interferon-associated retinopathy have been reported. Since the development of irreversible visual dysfunction has been reported, special attention to patients with comorbid hypertension and diabetes mellitus appears necessary [51]. Some recent studies have reported that extended maintenance TMZ does not contribute to PFS or OS [34, 52]. Balana et al. [53] reported that extended maintenance TMZ not only failed to improve PFS or OS, but also increased thrombopenia and lymphopenia. However, the present study encountered no severe adverse events such as interstitial pneumonia, and all observed adverse events (including hematological adverse events) were considered tolerable.

## Conclusions

In general, the adverse events of this study were tolerable, and 2y-OS of 40.7% as the primary endpoint was higher than that of the historical controls. However, the lower limit of the 95%CI was not exceed. Although a significant difference from historical controls was not able to be demonstrated, the treatment regimen applied may be promising. The results also suggest that MGMT methylation status may counteract the effects of PFS and OS. However, a well-designed, large-scale randomized controlled trial with biomolecular studies is required in the future.

## Data Availability

The datasets used and/or analyzed during the current study are available from the corresponding author on reasonable request.

## Abbreviations

CTCAE: Common Terminology Criteria for Adverse Events
CI: Confidence interval
C: Cycles
ECOG-PS: Eastern Cooperative Oncology Group performance scale
GBM: Glioblastoma
HGG: High-grade glioma
IFN-β: Interferon β
IDH: Isocitrate dehydrogenase
MGMT: O6-methyl-guanine-DNA-methyltransferase
mOS: Median overall survival
mPFS: Median progression-free survival
MS-PCR: Methylation-specific polymerase chain reaction
OS: Overall survival
PFS: Progression-free survival
RT: Radiotherapy
TMZ: Temozolomide
VCR: Vincristine
CCNU: Lomustine
ACNU: Nimustine
